# Fairness of recommender systems in the recruitment domain: an analysis from technical and legal perspectives

**DOI:** 10.3389/fdata.2023.1245198

**Published:** 2023-10-06

**Authors:** Deepak Kumar, Tessa Grosz, Navid Rekabsaz, Elisabeth Greif, Markus Schedl

**Affiliations:** ^1^Multimedia Mining and Search Group, Institute of Computational Perception, Johannes Kepler University Linz, Linz, Austria; ^2^Institute for Legal Gender Studies, Johannes Kepler University Linz, Linz, Austria; ^3^Human-centered AI Group, AI Lab, Linz Institute of Technology, Linz, Austria

**Keywords:** recommender system, recruitment, job recommendation, candidate recommendation, fairness, discrimination, law

## Abstract

Recommender systems (RSs) have become an integral part of the hiring process, be it via job advertisement ranking systems (job recommenders) for the potential employee or candidate ranking systems (candidate recommenders) for the employer. As seen in other domains, RSs are prone to harmful biases, unfair algorithmic behavior, and even discrimination in a legal sense. Some cases, such as salary equity in regards to gender (gender pay gap), stereotypical job perceptions along gendered lines, or biases toward other subgroups sharing specific characteristics in candidate recommenders, can have profound ethical and legal implications. In this survey, we discuss the current state of fairness research considering the fairness definitions (e.g., demographic parity and equal opportunity) used in recruitment-related RSs (RRSs). We investigate from a technical perspective the approaches to improve fairness, like synthetic data generation, adversarial training, protected subgroup distributional constraints, and *post-hoc* re-ranking. Thereafter, from a legal perspective, we contrast the fairness definitions and the effects of the aforementioned approaches with existing EU and US law requirements for employment and occupation, and second, we ascertain whether and to what extent EU and US law permits such approaches to improve fairness. We finally discuss the advances that RSs have made in terms of fairness in the recruitment domain, compare them with those made in other domains, and outline existing open challenges.

## 1. Introduction

Recommender systems (RSs) in the recruitment domain are usable by both job seekers (job recommenders) and candidate seekers (candidate recommenders). An early application of the recruitment-related RSs (RRSs) is CASPER (Case-Based Profiling for Electronic Recruitment) (Rafter et al., 2000), an automated collaborative filtering-based personalized case retrieval system. Most modern RRSs, such as LinkedIn, have diversified their approaches and use a variety of other methods such as exploiting textual data available in the recruitment domain, or social network knowledge (Fawaz, 2019; Geyik et al., 2019). In terms of algorithms, we have come a long way from linguistics-based systems (Vega, 1990) to the current RSs which are based on deep neural networks, collaborative filtering, content-based, and knowledge-based techniques (Gutiérrez et al., 2019; Bian et al., 2020; Gugnani and Misra, 2020; Lacic et al., 2020).

However, the prevalent use of RSs has also highlighted the possibility of biased outcomes in the recruitment domain. For instance, the gender stereotypes pertaining to particular professions are observed in the current workforce (Wilson et al., 2014; Smith et al., 2021). These stereotypes can further find their way into RRSs in the form of algorithmic bias (Tang et al., 2017; Ali et al., 2019; Raghavan et al., 2020). Algorithmic bias can cause discrimination in the exposure of the job advertisement or the algorithmic hiring itself. Facebook's advertisement delivery system, for example, suffered from algorithmic gender bias while showing job advertisements (Ali et al., 2019). The Amazon hiring algorithm, infamously favoring male over female job applicants, is another real-world example (Dastin, 2018). The historical data provided to the algorithm suggested that male applicants were preferred because previously more men than women had been hired. Such behaviors prompted the industry to adopt bias mitigation in RRSs (Raghavan et al., 2020).

Such algorithmic biases can have legal consequences. Hiring decisions, whether algorithmically assisted through RRSs or solely taken by the employer, are part of the employment process and as such do not operate in a legal vacuum: Non-discrimination law plays an essential role in safeguarding from discrimination in the recruitment domain and should not be overlooked by RRS researchers. Given this context, the objective of this survey is threefold:

- to examine recent studies focusing on fairness in RRSs,
- to emphasize the significant disparities between the research conducted in the fields of computer science (CS) and law concerning this topic, and
- to identify the challenges that exist within fairness research for RSs employed in the recruitment domain.

### 1.1. Related surveys

There exist multiple surveys on the fairness of RSs (Zehlike et al., 2022; Deldjoo et al., 2023; Wang et al., 2023). While these surveys partly covers work on RRSs, they are not tailored to this domain, nor do they connect the discussion of fairness to the legal aspects of this domain. These two points are crucial, particularly due to the essential differences of RRSs from those used in other domains (e.g., video, music, or e-commerce): First, the decision of the system may have significant impacts on the end-users in terms of fairness and distribution of resources and can have serious legal implications. Second, the recruitment domain heavily relies on textual data of partly personal and sensitive nature, for instance, the resumes, and job posts which can contain sensitive information about candidates and employers, respectively. There also exist several surveys on RRSs, e.g., Dhameliya and Desai (2019), de Ruijt and Bhulai (2021), Freire and de Castro (2021), and Thali et al. (2023), but only de Ruijt and Bhulai (2021) include a dedicated section covering fairness aspects. However, de Ruijt and Bhulai (2021) provide a broad scope on the topic, and only present high-level insights on fairness aspects. In particular, we are not aware of any survey in this domain that draws the connection between algorithmic aspects of RRSs and the intertwined legal facets. To fill these gaps in existing surveys on the fairness of RSs and RRSs, the survey at hand calls for attention to the research specifically addressing the fairness of RSs in the recruitment domain, with a multidisciplinary analysis from both technical and legal perspectives.

### 1.2. Literature search

To identify relevant literature, we conducted a series of searches on DBLP^1^ with the keywords “Job”/“Candidate” in conjunction with “Fair”/“Bias” to create a candidate list of publications for this survey. Subsequently, we selected a subset of this list based on their relevance to RSs in the recruitment domain after a careful manual inspection of each paper. We further enriched the list with articles from the workshop series on Recommender Systems for Human Resources (RecSys in HR) and with further recent works spotted by studying the references in the collected papers. The surveyed literature covers the work published until May 2023.

### 1.3. Outline

The survey is structured as follows: Section 2 provides an overview of the current research on fairness in RRSs. In Section 3, we focus on the significant legal aspects that pertain to RSs in the recruitment domain, aiming to bridge the gap between the legal and technical dimensions of ensuring fairness in candidate recommenders, and to highlight the shortcomings of the existing approaches for fairness in RS. Finally, Section 4 discusses the open challenges and future directions related to fairness research in RRSs. For easy reference, Table 1 contains a list of abbreviations used throughout the article.

**Table 1:** List of abbreviations used in the article. For abbreviations of fairness metrics, please refer to Table 3.

AI	Artificial Intelligence
CBF	Content-based Filtering
CF	Collaborative Filtering
CJEU	Court of Justice of the European Union
CS	Computer Science
DP	Demographic Parity
EO	Equal Opportunity
IF	Individual Fairness
KB	Knowledge-Based
PF	Proportional Fairness
RRS	Recruitment-related Recommender System
RS	Recommender System

## 2. Current state of research

As a core reference for interested readers, we provide in Table 2 an overview and categorization of existing research that addresses fairness in RRSs. The individual works are classified based on the datasets utilized for the experiments, the aspired fairness definitions, the metrics employed to assess fairness, the stage in the pipeline where debiasing strategies are implemented to achieve fairness, and the sensitive attributes explored during the experiments.

**Table 2:** Overview and categorization of the research works surveyed.

**Article**	**RS**		**Dataset(s)**	**Fairness measurement**		**Debiasing strategy**	**Sensitive attribute(s)**
	**Type**	**Algorithm**		**Definition(s)**	**Metric(s)**		
Li et al. (2023)	Job	CF	Private	DP	UGF	Post	Gender (G)
Rus et al. (2022)	Job	CBF	Private	EO, DP	TPRP, SAT	Pre, In	G
Ntioudis et al. (2022)	Job	KB	Synthetic, private	—	—	Pre	Migrant
Zhang (2021)	Job	—	Synthetic	DP	SDR, LDR	—	G
Shishehchi and Banihashem (2019)	Job	KB	Private	—	UAT	Pre	Disability
Tang et al. (2017)	Job	—	Scraped	—	MWU	—	G
Scher et al. (2023)	Candidate	—	Synthetic	EO, DP	BGSD, CF, TNR	Post	—
Jourdan et al. (2023)	Candidate	CBF	BIOS	EO	TPRP	Pre, In	G
Delecraz et al. (2022)	Candidate	—	Private	EO	TPRP, DI, SP	—	Multi
Markert et al. (2022)	Candidate	CBF	Synthetic	IF	IFC	—	G, Marital status
Bei et al. (2020)	Candidate	Other	Synthetic	PF	Violations	Post	G, Region
Arafan et al. (2022)	Candidate	—	Synthetic, Private	DP	NDKL	Pre, Post	G
Tran et al. (2022)	Candidate	KB	ACI, TO, HR	—	—	Post	Disability
Syed and Shivendu (2022)	Candidate	—	—	EO, DP	TPRP	Pre	—
Burke et al. (2021)	Candidate	Other	SIOP2021	EO	AIR	Post	—
Tran et al. (2021)	Candidate	KB	Synthetic, Private	—	—	Post	Disability
Wilson et al. (2021)	Candidate	—	Private	EO, DP	AIR, MWU, KWH	—	G, Ethnicity
Elbassuoni et al. (2020)	Candidate	—	Synthetic, Private	DP	EMD	—	G, Ethnicity
Elbassuoni et al. (2019)	Candidate	—	Private	DP	EMD	—	Multi
Geyik et al. (2019)	Candidate	—	Synthetic, Private	EO, DP	NDKL, MS, II, IC	Post	G, Age
Chen et al. (2018)	Candidate	—	Scraped	DP, IF	MWU, TDRC, EC	—	G
Amer-Yahia et al. (2020)	Both	—	Synthetic, Private	DP	KT, EMD, Exposure	—	G, Ethnicity, Nationality

In RS Type, “Both” is used when both job recommender and candidate recommender are used, and for Sensitive Attribute(s), “Multi” is used when there are four or more attributes (e.g., Gender, Nationality, Age, Ethnicity, Language). The symbol “—” is used when the column is not applicable or cannot be inferred from the paper.

This section analyzes the current state of fairness research in RRSs. Our examination of the existing literature is structured around various aspects, including the *recommendation algorithm* underlying the RRSs (Section 2.1), the *datasets* used in the reviewed works (Section 2.2), the *definition of fairness* (Section 2.3), the *metrics* used to evaluate fairness or unfairness in the datasets and created recommendations (Section 2.4), *evidence of bias* within these algorithms (Section 2.5), and the different *approaches* explored to attain fairness (Section 2.6).

In the article at hand, we adopt the terminology common in RSs research, i.e., we refer to “users” (*U*) and “items” (*I*), the former representing the entity for which recommendations are made (input), the latter the recommendations themselves (output). Hence, for a job recommender system, the user is the candidate looking for a job, and the items are job posts. In contrast, for a candidate recommender, the user is the job post and the items are candidates.

### 2.1. Recommendation algorithms in the recruitment domain

RRSs can be classified into four categories: collaborative filtering (CF), content-based filtering (CBF), knowledge-based (KB) recommenders, and hybrid recommenders. In research on fairness of RRSs, company platforms are mostly investigated, e.g., Indeed, Monster, and CareerBuilder^2^ in Chen et al. (2018); LinkedIn in Tang et al. (2017) and Geyik et al. (2019); TaskRabbit and Google Job Search^3^ in Amer-Yahia et al. (2020) and Elbassuoni et al. (2020). These works are about investigating fairness of these popular platforms rather than improving it.

In contrast to the proprietary and commonly non-disclosed recommendation algorithms used by the aforementioned services, RRS approaches found in the surveyed literature can be categorized into the following:

- Collaborative Filtering (CF): CF algorithms employed in RRSs are all based on the similarity between users, calculated through the users' interactions with the items. In the RRSs literature, users are always job seekers, i.e., no CF approaches are used for candidate recommenders, only job recommenders. The adopted algorithms include probabilistic matrix factorization (Salakhutdinov and Mnih, 2007), neural matrix factorization (He et al., 2017), session-based model STAMP (Short-Term Attention/Memory Priority) (Liu et al., 2018), and matrix factorization with global bias terms (Koren et al., 2009).
- Content-based Filtering (CBF): CBF algorithms are based on the similarity between the items and the users, most commonly implemented as direct matching of users and items through text-based similarity. In the surveyed RRSs literature, users can be job posts (candidate recommenders) or candidates (job recommenders). Rus et al. (2022) and Jourdan et al. (2023) implement RoBERTa (Liu et al., 2019) and BERT (Devlin et al., 2019) language models, respectively, to learn candidate-job similarity. Markert et al. (2022) use a custom regression model to learn the candidate-job similarity. In these works, both language model and regression model are neural network-based models.
- Knowledge-based (KB): The algorithms in this category utilize domain-specific knowledge to create ontology and compute similarity between user-item pairs. Existing works that fall in this category use directed acyclic causal graphs (Tran et al., 2021, 2022) and knowledge representation using domain ontology (Shishehchi and Banihashem, 2019; Ntioudis et al., 2022).
- Hybrid: Hybrid approaches utilize combinations of the previous approaches (Luo et al., 2019). No such hybrid could be found in the conducted literature search.
- Others: We group here the studies that do not use the mentioned recommendation approaches. In particular, Burke et al. (2021) implements spatial search-based candidate selection, and Bei et al. (2020) deploys integer linear programming-based candidate selection.

### 2.2. Datasets

In Table 2, we divide the datasets into four categories: dataset that cannot be accessed or recreated (denoted *Private* in the table), scraping details are given (*Scraped*), procedure to create the artificial dataset is given (*Synthetic*), and name of the dataset if it is public. The datasets used in the reviewed works are primarily private or synthetically created. Table 2 also reveals the surprising absence of popular public job recommender datasets, such as the datasets used in the ACM Recommender Systems Challenge 2016 and 2017 (Abel et al., 2016, 2017) and the Career Builder 2012 dataset.^4^ Public datasets are used only for candidate recommenders.

- *BIOS (De-Arteaga et al.*, *2019**):* This dataset has been created by scrapping biographies using Common Crawl.^5^ The dataset contains biographies of individuals with the attributes current job and gender. The dataset proposed by the authors contains 397,340 biographies with 28 different occupations.
- *Adult Census Income (ACI) (Becker and Kohavi*, *1996**):* The ACI dataset has been extracted from the 1994 USA census database and includes 48,842 individuals' records. For each individual, it contains features such as occupation, age, education, marital status, salary, race, and gender.
- *TO*^6^: The dataset contains 1,129 Russian workers' income and 16 features describing them, such as gender, age, profession, experience, industry, employee turnover, supervisor, supervisor's gender, and recruitment route.
- *HR*^7^: This dataset contains 15,000 individuals' retention records with the features satisfaction level, last evaluation, average monthly hours, work accident, salary, and time spent in company.
- *SIOP2021 (Koenig and Thompson*, *2021**):* This dataset was introduced in the machine learning challenge of the Annual Conference of the Society for Industrial and Organizational Psychology 2021. The dataset contains the three attributes performance, turnover data, and the protected group membership^8^ for 7,890 respondents.

### 2.3. Fairness definitions for recruitment-related recommender systems

We review different fairness definitions in RRSs in this section. Overall, as shown in Table 2, one of the most used fairness definitions in the context of RRSs is demographic parity (DP). Also, the fairness definition for job recommenders is restricted to DP in the identified literature. Overall, DP is the most often used fairness definition for both job and candidate recommenders. We will see in the definition of DP that it does not consider the quality of recommendation (i.e., whether relevant items are ranked high or low is ignored. This results in an easier adaptation of this fairness definition from classification to recommendation tasks for both candidate recommenders and job recommenders.

Most fairness definitions mentioned in the literature are adapted from the binary classification setting to the RS setting. The core fairness definitions from the classification are listed below:

- *Demographic parity (DP):* A binary predictor *Ŷ* is said to satisfy demographic parity with respect to protected attribute *A*∈{*a*_1_, ..., *a*_*l*_} that can take *l* values if *Ŷ* is independent of *A* (Dwork et al., 2012).
$$P\left(Ŷ|A=a_{1}\right)=...=P\left(Ŷ|A=a_{l}\right)$$
- *Equal opportunity (EO):* A binary predictor *Ŷ* is said to satisfy equal opportunity with respect to a protected attribute *A*∈{*a*_1_, ..., *a*_*l*_} that can take *l* values and ground truth *Y* if they are independent conditional on the ground truth outcome being favorable (Hardt et al., 2016).
$$P\left(Ŷ=1|A=a_{1},Y=1\right)=...=P\left(Ŷ=1|A=a_{l},Y=1\right)$$
- *Individual fairness (IF):* A predictor *Ŷ* is said to satisfy individual fairness if for data-point *x* from dataset *D* for all *x*′ that are similar to *x* (i.e., all their attributes are the same except for the sensitive attribute) the predictor predicts the same class (Ruoss et al., 2020).
$$x\sim D,\forall x^{\prime }\in \mathbb{R}:\phi \left(x,x^{\prime }\right)\;\Rightarrow \;\mu \left({Ŷ}_{x},{Ŷ}_{x^{\prime }}\right)$$
  where ϕ(*x, x*′) = 1 iff *x* and *x*^′^ are similar
  and $\mu \left({Ŷ}_{x},{Ŷ}_{x^{\prime }}\right)=1\Leftrightarrow {Ŷ}_{x}={Ŷ}_{x^{\prime }}$

As we explain in the following, the fairness definitions for candidate recommenders are strongly aligned with classification scenarios, i.e., whether an item (candidate) occurs in the recommendation list or not. At the same time, fairness definitions for job recommenders require further modification. The fairness definitions in RRSs are always from the candidate's perspective. In the case of job recommenders, the involved variables are sensitive attributes *A*_*u*_ (e.g., gender or ethnicity) of the input *u* (i.e., candidate), and the recommended list *Q*_*u*_ (i.e., list of job posts). For candidate recommenders, the sensitive attribute *A*_*i*_ belongs to the item *i* in the recommended list *Q*_*u*_ (i.e., list of candidates). Furthermore, defining fairness for job recommenders requires the function F defined over the recommended list *Q*_*u*_ (list of job posts), which measures the quality of recommendation (e.g., through precision or recall metrics) or some other property of *Q*_*u*_ like the average salary of jobs in *Q*_*u*_. Based on these definitions, in the following, we review the fairness definitions for RRSs, adapted from classification tasks:

- *DP for job recommender (Li et al.*, *2023**)*: A job recommender satisfies DP for sensitive attribute gender *A*_*u*_∈{*male, female, non*−*binary*} if some measure, expressed as a function F defined over the recommendation list of jobs, is independent of the value of *A*_*u*_, i.e.,
$$\begin{array}{r} P\left(F\left({\text{Q}}_{u}\right)|A_{u}=male\right)=P\left(F\left({\text{Q}}_{u}\right)|A_{u}=female\right)\\ =P\left(F\left({\text{Q}}_{u}\right)|A_{u}=non-binary\right) \end{array}$$
  Example: The average salary of jobs recommended have the same distribution for the male, female, and non-binary candidate groups.
- *DP for candidate recommender (Geyik et al.*, *2019**)*: A candidate recommender satisfies DP for attribute tuple *A*_*i*_ = < *gender*_*i*_, *age*_*i*_>, if the existence or absence of any candidate *i* in the recommended list *Q*_*u*_ for job ad *u* is independent of their attributes *A*_*i*_.
$$\begin{array}{r} P(i\in Q_{u}|A_{i}=<gender_{i},age_{i}>)=P(j\in Q_{u}|A_{j}\\ =<gender_{j},age_{j}>)\\ P(i\notin Q_{u}|A_{i}=<gender_{i},age_{i}>)=P(j\notin Q_{u}|A_{j}\\ =<gender_{j},age_{j}>) \end{array}$$
  Example: A young male has the same probability as an old female of being included in the recommendation list for the job.
- *EO for candidate recommender (Geyik et al.*, *2019**)*: A candidate recommender satisfies EO with respect to protected attribute tuple *A*_*i*_ = < *gender*_*i*_, *age*_*i*_> if candidate *i*'s existence in the recommended list *Q*_*u*_ with respect to protected attributes *A*_*i*_ is conditionally independent of the candidate being qualified [i.e. ρ(*i, u*) = 1] for the job *u*.
$$\begin{array}{l} P\left(i\in Q_{u}|A_{i}=<gender_{i},age_{i}>,\rho \left(i,u\right)=1\right)\\ =P\left(j\in Q_{u}|A_{j}=<gender_{j},age_{j}>,\rho \left(i,u\right)=1\right) \end{array}$$
  Example: A young male who satisfies the requirements for the job has the same probability of being selected for the job as an old female that satisfies the same requirements.
- *IF for candidate recommender (Markert et al.*, *2022**)*: A candidate recommender satisfies IF if for an item *i* (i.e., candidate) in the recommended list *Q*_*u*_ (for a job ad *u*) all the candidates *i*′ similar to *i* (i.e., their attributes are the same except for the sensitive attribute) in the recommended lists have nearby positions in the ranking.
$$i\in Q_{u},\forall i^{\prime }\in Q_{u}:\phi \left(i,i^{\prime }\right)\;\Rightarrow \;\mu \left(Q_{u},i,i^{\prime }\right)$$
  where ϕ(*i, i*′) = 1 iff *i* and *i*^′^ are similar
  and $\mu \left(Q_{u},i,i^{\prime }\right)=1\Leftrightarrow pos\left(i,Q_{u}\right)\approx pos\left(i^{\prime },Q_{u}\right)$
  Example: If two candidates of different gender in the item set have the same attributes, they should be ranked at nearby positions in the recommendation list.

In contrast to the mentioned definitions, *Proportional Fairness (Bei et al.*, *2020**)* is not adapted from classification and is directly defined for RSs.

- *Proportional Fairness (PF) (Bei et al.*, *2020**)*: For a candidate recommender to satisfy PF, the selected set of candidates *S* and candidate attribute set *A* = {*a*_1_, ..., *a*_*l*_}, at least fraction α_*j*_ and at most fraction β_*j*_ of *S* have attribute *a*_*j*_.
  Example: Five candidates are selected for a given job by the RRS. The attributes of these people are gender (male vs. female) and region (Europe vs. Africa). Then, for proportional fairness over the attributes male, female, Europe, and Africa under the constraints (least fraction, most fraction), respectively, (0.4, 0.6), (0.4, 0.6), (0.2, 0.4), and (0.2, 1.0), it is required that the number of people out of 5 with attribute male, female, Europe, and Africa are {2, 3},{2, 3},{1, 2}, and {1, 2, 3, 4, 5}, respectively.

### 2.4. Fairness and unfairness metrics

Formalizing the fairness definitions introduced above, the RRSs literature has proposed various metrics to quantify the degree to which fairness is achieved by a given RRS. In this section, we therefore present the fairness metrics used in the surveyed literature. As we could already see in Table 2, they are very diverse. The only recurring metrics are TRPR, EMD, NDKL, MWU, and AIR. The frequency of each metric's use in literature can be seen in Table 3, along with a categorization of the metrics with respect to the targetted fairness definitions. Below we introduce the common metrics and the metrics we refer to later in the manuscript. For the remaining ones, we refer the reader to the corresponding references provided in Table 2.

**Table 3:** Metrics categorized according to the fairness definition associated, with frequency of papers using the metric.

**Fairness definition**	**Metric**	**Frequency**
Demographic Parity (DP)	Earth Mover's Distance (EMD)	3
	Mann-Whitney U test (MWU)	3
	Normalized KL Divergence(NDKL)	2
	User Group Fairness (UGF)	1
	Salary Association Test (SAT)	1
	Set Difference Rate (SDR)	1
	List Difference Rate (LDR)	1
	Min/Max Skew (MS)	1
	Kendall's Tau (KT)	1
	Kruskal-Wallis H test (KWH)	1
	Total Difference between the Recall Curves (TDRC)	1
	Equal opportunity (EO)	True Positive Rate Parity (TPRP)	4
Adverse Imact Rate (AIR)		2
Counterfactual Fraction (CF)		1
True Negative Rate (TNR)		1
Infeasible Index (II)		1
Infeasible Count (IC)		1
Exposure		1
Individual Fairness (IF)	Individual Fairness Certification (IFC)	1
	Effect Coefficient (EC)	1
Proportional Fairness (PF)	Violations	1
—	User Acceptance Test (UAT)	1
	Between Group Skill Difference (BGSD)	1
	Disparate Impact (DI)	1
	Statistical Parity (SP)	1

The metrics in the literature addressing DP consider the distribution of sensitive attributes over recommended lists, as shown for here:

- *User Group Fairness (UGF) (Li et al.*, *2023**)*: Given metric F (e.g., average salary) over a recommended list of jobs *Q*_*u*_ for the user *u* (i.e. candidate) and the set of all candidates *U* partitioned in two mutually exclusive sets *U*_*a*_1__, *U*_*a*_2__ based on protected attribute *A*∈{*a*_1_, *a*_2_}, UGF is defined as:
$$UGF\left(U_{a_{1}},U_{a_{2}},Q\right)=|\frac{1}{\left|U_{a_{1}}\right|}\underset{u\in U_{a_{1}}}{\sum }F\left({\text{Q}}_{u}\right)-\frac{1}{\left|U_{a_{2}}\right|}\underset{u\in U_{a_{2}}}{\sum }F\left({\text{Q}}_{u}\right)|$$
  where *Q* = {*Q*_*u*_∀*u*∈*U*} is the set of all recommendation lists.
- *Set Difference Rate (SDR) (Zhang*, *2021**)*: SDR is defined to measure proportion of attribute-specific jobs. SDR between set of items (i.e., jobs) *I*_*a*_1__ and *I*_*a*_2__ recommended only to users (i.e., candidates) with sensitive attribute *A* = *a*_1_ and *A* = *a*_2_ (where *A*∈{*a*_1_, *a*_2_}) respectively and the set of all jobs *I*.
$$SDR\left(I_{a_{1}},I_{a_{2}}\right)=\frac{\left|I_{a_{1}}|+|I_{a_{2}}\right|}{\left|I\right|}$$
- *List Difference Rate (LDR) (Zhang*, *2021**)*: LDR is defined to measure differences in ranking due to the binary protected attribute *A*∈{*a*_1_, *a*_2_} of a user. LDR for a pair of recommendation lists *Q*_*u*_ and *Q*_*û*_ of a candidate *u* and its counterfactual *û* (created by changing *u*'s protected attribute), respectively, is
$$LDR\left(Q_{u},Q_{û}\right)=\frac{\overset{\left|Q_{u}\right|}{\underset{j=1}{\sum }}\gamma \left(Q_{u},Q_{û},j\right)}{\left|Q_{u}\right|}$$
  where γ(*Q*_*u*_, *Q*_*û*_, *j*) = 1if the*j*^th^ job ad in *Q*_*u*_ and *Q*_*û*_ is the same.
- *Earth Mover's Distance (EMD) (Pele and Werman*, *2009**)*: EMD can be defined for sensitive attribute *A*∈{*a*_1_, ..., *a*_*l*_} that can take *l* values by dividing the ranking *Q*_*u*_ in two groups, group *G* with *A* = *a*_1_ and group *Ḡ* with *A* ≠ *a*_1_. EMD between the two groups represents the smallest amount of required change in the ranking scores of the bigger group to obtain the ranking scores of the smaller group. Then, EMD between *G* and *Ḡ* for the smallest amount of change in the ranking score scenario is
$$EMD\left(G,Ḡ\right)=\frac{\sum_{i,j}f_{i,j}|i-j|}{\sum_{i,j}f_{i,j}}$$
  where *f*_*i, j*_ is the change in *i*^th^ rank score of the bigger group to get the *j*^th^ rank score of the smaller group. To give an example, assume that the two groups are young *G* and old *Ḡ* candidates. Groups are represented by the score their members have at each rank. So, for *G* = {0, 0.3, 0.2} and *Ḡ* = {0.5, 0, 0}, the solution of smallest change is to rerank *G* to get the same score distribution as *Ḡ*. Here, $EMD\left(G,Ḡ\right)=\frac{0.3*1+0.2*2}{0.3+0.2}=1.4$.
- *Normalized Discounted Kullback-Leibler Divergence (NDKL) (Geyik et al.*, *2019**)*: Given ranked list *Q*_*u*_ and two distributions $P_{A_{Q_{u}}}$ and $P_{A}$, which are distributions of attribute *A* in *Q*_*u*_ and the desired distribution of *A*, respectively, the NDKL is
$$\begin{array}{l} NDKL\left(Q_{u}\right)=\frac{1}{Z}\overset{\left|Q_{u}\right|}{\underset{j=1}{\sum }}\frac{1}{log_{2}\left(j+1\right)}d_{KL}\left(P_{A_{Q_{u}}}||P_{A}\right),\\ Z=\overset{\left|Q_{u}\right|}{\underset{j=1}{\sum }}\frac{1}{log_{2}\left(j+1\right)} \end{array}$$
  where *d*_*KL*_ is KL-divergence. It should be noted that *NDKL* = 0 for all users would imply demographic parity is achieved.
- *Mann-Whitney U (MWU) test (Corder and Foreman*, *2014**)*: MWU test is a non-parametric statistical test where the null hypothesis for job recommender is:
  • *For candidate recommender*: The ranks of candidates with a particular protected attribute is not significantly different from the ranks of candidates with another attribute.
  • *For job recommender*: The recommended list of job posts is not significantly different for candidates with different protected attributes.

The metrics associated with EO are dependent on the quality of recommendations and the distribution of sensitive attributes over recommended lists.

- *True Positive Rate Parity (TPRP) (Delecraz et al.*, *2022**)*: TPRP in candidate recommendation for a given binary protected attribute *A*∈{*a*_1_, *a*_2_} and recommendation list *Q*_*u*_ for job *u* is defined as
$$\begin{array}{l} TPRP\left(u\right)=|P\left(x\in Q_{u}|A_{x}=a_{1},\rho \left(u,x\right)=1\right)\\ -P\left(x\in Q_{u}|A_{x}=a_{2},\rho \left(u,x\right)=1\right)| \end{array}$$
  where ρ(*u, x*) = 1 implies that a candidate *x* sampled randomly from the candidate set is suitable for job *u*, and *TPRP* = 0 implies EO is achieved. TRPR is also called sourcing bias (Syed and Shivendu, 2022) and true positive rate gap (Jourdan et al., 2023) in the literature.
- *Adverse Impact Ratio (AIR) (Burke et al.*, *2021**)*: AIR for binary sensitive attribute *A*∈{*a*_1_, *a*_2_} and recommended list *Q*_*u*_ for job *u* is,
$$AIR\left(u\right)=\frac{\left|P\left(x\in Q_{u}|A_{x}=a_{1},\rho \left(u,x\right)=1\right)\right|}{\left|P\left(x\in Q_{u}|A_{x}=a_{2},\rho \left(u,x\right)=1\right)\right|}$$
  where, ρ(*u, x*) = 1 implies that a candidate *x* sampled randomly from the candidate set is suitable for job *u*, and *AIR* = 1 implies EO is achieved.

There also exist a few metrics which are not associated with a particular fairness definition but are nevertheless related with fairness, e.g., UAT measures the disadvantaged groups' perception of the RRS, and several metrics quantify the fairness of RRS datasets.

- *User Acceptance Test (UAT) (Shishehchi and Banihashem*, *2019**)*: UAT is a questionnaire to assess the quality of job recommenders for people with disability, used by the authors in a user study. The questionnaire investigates four factors, i.e., usefulness, ease of use, ease of learning, and satisfaction with the RRS. The responses are registered in terms of values from 1 (disagree) to 5 (agree) for questions related to each factor.
- *Dataset Bias Metrics (Delecraz et al.*, *2022**)*: Statistical parity (SP) and Disparate impact (DI) metrics are used for measuring the bias in datasets with respect to binary attribute *A*∈{*a*_1_, *a*_2_}. Here, ρ(*u, x*) = 1 denotes that candidate *u* is qualified for a job post *x* randomly sampled from the set of job posts:
$$SP(u)=P(\rho (u,x)=1|A=a_{1})-P(\rho (u,x)=1|A=a_{2}))$$
$$DI(u)=\frac{P(\rho (u,x)=1|A=a_{1})}{P(\rho (u,x)=1|A=a_{2})}$$

### 2.5. Investigating fairness of recommendations

A subset of reviewed articles analyzes fairness in RRSs. Tang et al. (2017) examines 17 million LinkedIn job listings spanning over 10 years and conducts a user study to analyze perceived stereotypes in these listings. The authors use the recruitment assistance services company Textio^9^ and Unitive (no longer operational) to get a list of gendered words and then, based on the weighted frequency of those words in a job listing, measure their “maleness” and “femaleness.” They find that job listings perceived as overall male and the usage of gendered words, in general, have decreased over the years. These results could suggest that our society is moving toward more gender-appropriate language. They also conducted a user study where two of the questions are “While reading the job description, to what extent did you feel that the advertisement would attract more male or more female applicants?” and “If you were fully qualified to apply for a job like this, how likely is it that you would apply for this particular position?” They compare user responses with the “genderedness” of job listings measured earlier using the Mann-Whitney U (MWU) test and found that there is a low correlation between the gendered wording of job listings and perceived gender bias (attractiveness to female applicants). Instead, the perceived bias depends on preconceived notions like technology jobs are male jobs or lower wage jobs are female jobs. Additionally, the willingness to apply had a low correlation with perceived gender bias or the gendered wording of job listing.

Similarly, Chen et al. (2018) investigates gender bias on Indeed, Monster, and CareerBuilder resume searches. They use a regression model to measure IF and DP using MWU. The IF for the candidates occurring at ranks 30 − 50 in the recommendation list shows that men occupy higher ranks compared to women, which can seem counter-intuitive. For DP, this advantage is significant for multiple job titles (e.g., Truck Driver and Software Engineer).

The work of Delecraz et al. (2022) analyzes bias of different attributes like age, gender, geography, and education in their private dataset with disparate impact (DI) and statistical parity (SP). They found that their dataset is fair along gender and age attributes but is unfair according to nationality. They use true positive rate parity (TPRP) to analyze EO over their private candidate recommender. They found that education, birthplace, and residence permit were impactful for the candidate selection of individuals, while age and gender were not. This is partially explained by the fact that education, birthplace, and residence permit, in many cases, can be seen as requirements rather than biases.

Elbassuoni et al. (2019, 2020) propose new heuristic- and decision-tree-based approaches, respectively, to find a partition of candidates based on their attributes for which unfairness in terms of DP is maximized. After partitioning, the authors use EMD to measure the unfairness in the ranked list. Then, following a similar methodological approach for measuring bias, Amer-Yahia et al. (2020) investigate the online recruitment platform TaskRabbit and the job recommender platform Google Job Search. The uniqueness of Elbassuoni et al. (2019, 2020) and Amer-Yahia et al. (2020) is that the authors consider partitioning based on combinations of attributes rather than single attributes.

Wilson et al. (2021) conduct a fairness audit of the Pymetrics candidate screening system.^10^ Through this audit, the authors try to verify Pymetrics' claim to abide the 4/5^th^ rule from the US Union Guidelines on Employee Selection Procedures (Cascio and Aguinis, 2001). According to the 4/5^th^ rule, if the selection rate of a group is less than 4/5^th^ of the highest group selection rate, then that group is adversely impacted. This rule closely aligns with the DP definition of fairness.

Zhang (2021) provides a gender fairness audit of four Chinese job boards. On these job boards, there are some job posts with explicit mention of preferred gender. The audit using list/set difference rate (LDR/SDR) showed the existence of gender bias in terms of quality of recommendation, and also differences in the wording of job ads recommended to males versus those recommended to female job seekers.

Markert et al. (2022) is the only article to pursue individual fairness (IF) and adapt the classification IF certification (IFC) process for ranking. The IF definition for candidate recommenders in Section 2.3 requires similar candidates to have similar ranks. A regression model is trained to predict a candidate's rank in the recommended list given by the candidate recommender. The authors formulate a mixed integer linear programming problem using the regression model and the similarity constraint to get upper and lower bounds for the output of the regression model, i.e., the candidate's rank.

### 2.6. Pre-, in-, and post-processing approaches for fairness

The approaches to achieve fairness in RRSs can be categorized into pre-, in-, and post-processing techniques.

*Pre-processing* approaches are applied to the training data of the RS. The following approaches are used in the literature on RRSs:

- *Balancing the dataset*: Balancing the training data with respect to the sensitive attribute. For instance, Arafan et al. (2022) create gender-balanced synthetic data using CTGAN (conditional tabular generative adversarial network) for training (Xu et al., 2019), resulting in a significant decrease in NDKL.
- *Replacing the pronouns*: A simple approach for gender bias mitigation is to replace gendered pronouns with gender-neutral pronouns, as performed for instance in Rus et al. (2022) and Jourdan et al. (2023). This approach shows no change in terms of TPRP compared to not using pronoun substitution for Rus et al. (2022), while Jourdan et al. (2023) show a slight improvement in TPRP. The difference in results could be due to the different language models used.
- *Constrained resume sourcing*: Syed and Shivendu (2022) find conditions regarding the number of relevant candidates in each subgroup at the data sourcing of resumes for training to reduce the TRPR and theoretically achieve EO and DP.
- *Special group RSs*: The ontology-based/KB job recommenders use the sensitive information (e.g., disability, age, location, language) as input to create dedicated RSs for special groups (e.g., migrants or disabled people) (Shishehchi and Banihashem, 2019; Ntioudis et al., 2022). Shishehchi and Banihashem (2019) show strong acceptance of their RS by the special group (disabled people) using UAT, while Ntioudis et al. (2022) do not evaluate their system for the special group (migrant people).

*In-processing* approaches to mitigate bias change the RS itself to make the recommendations less biased. The in-processing approach is the least used method in the RRS literature. Also, in-processing approaches are only used with CBF.

- *Adversarial debiasing*: Rus et al. (2022) fine-tune a large language model to learn job-candidate similarity with the additional objective of removing gender information from the embedding of job posts, using an adversarial network that tries to predict the gender of the candidate. The adversarial debiasing shows significant improvement in TPRP compared to replacing pronouns.
- *Regularization-based debiasing*: Methods such as Jourdan et al. (2023) use a regularization term in the loss. network. In this case, the regularization term is the Sinkhorn Divergence (Chizat et al., 2020) over the distribution of sensitive attributes in the predictions. Similar to adversarial debiasing, this approach shows significant improvement in TPRP compared to replacing pronouns.

*Post-processing* approaches are applied to the ranking received from the RRS to re-rank the items (jobs or candidates in our case). In research on fairness of RRSs, post-processing approaches are the most common, then pre-processing approaches, and in-processing are least explored in the literature (see Table 2), which is strikingly different from the fairness research in RSs overall (Deldjoo et al., 2023), where this order is in-, post-, and pre-processing, respectively. This comparison should be seen with caution though, as the number of papers surveyed is significantly less here compared to Deldjoo et al. (2023). The following are the post-processing approaches used in the RRSs we identified:

- *Introducing proportional fairness constraints*: Bei et al. (2020) try to achieve PF by removing the lowest ranking candidate inside the recommended list for which the attributes' upper bound condition (i.e., β) of PF gets violated, and adding the highest ranking candidate outside the recommended list for which the attributes' lower bound condition (i.e., α) of PF gets violated. This approach is able to approach PF with very few constraints violated.
- *Deterministic constrained sorting*: Geyik et al. (2019) introduce a deterministic sorting algorithm with constraints similar to the PF constraints. The difference with the work of Bei et al. (2020) is that here each candidate has only one attribute rather than a set of attributes and the size of the recommended list is not fixed here. Arafan et al. (2022) re-rank the recommended list to achieve the same number of candidates for each attribute using the re-ranking algorithm of Geyik et al. (2019). Both papers show improvement in NDKL. Arafan et al. (2022) additionally show that the NDKL scores of the deterministic constrained sorting approach can be further improved by using an artificially balanced dataset.
- *Spatial partitioning*: Burke et al. (2021) introduce a re-ranking algorithm based on spatial partitioning of 3-dimensional space created by three attributes of the candidate, i.e., performance, retention after hiring, and whether the candidate belongs to a protected group or not. Improvements in terms of AIR score are mentioned by Burke et al. (2021) though scores are not reported.
- *Targeting user group fairness*: Li et al. (2023) re-rank the recommended list of jobs for all candidates to maximize the sum of each user's personalized utility score (Zhang et al., 2016) over all candidate-job pairs while minimizing the UGF value.
- *Intervention-based skill improvement*: The method proposed by Scher et al. (2023) selects candidates to upgrade their skills so that it improves their probability of selection by the candidate recommenders. The authors divide the candidates into high-prospect group and low-prospect group using their skills and sensitive information as decision criteria. Then, the high-prospect groups' skill is upgraded and the skill upgrade of the low-prospect group is delayed for some time. The method helps the high-prospect group and punishes the low-prospect group, resulting in less reduction of inequality (i.e., less improvement in EO and DP) in the long run compared to random selection of candidates. And the selection of only low-prospect group has no impact on EO in the long run.
- *Attribute intervention*: Tran et al. (2021) and Tran et al. (2022) identify the skill set of the candidate that should be upgraded and the level to which it should be upgraded based on candidate attributes (including protected attributes) to achieve improvement in selection probability by a causal tree-based candidate recommender, more precisely, by a maximal causal tree (Tran et al., 2021) or personalized causal tree (Tran et al., 2022).

The reviewed debiasing approaches work toward fairer RRSs from a technical perspective, but the recruitment domain requires to take a legal perspective too since obligations and requirements from employment law apply. In the next section, we will therefore give an overview of the most critical legal pitfalls for RRSs. With this, we aim at giving the researchers and developers of RRSs guidelines on possible legal issues of their systems.

## 3. Legal validity

When RSs are used in the hiring process to (help) make decisions, the legal requirements from employment law are applicable to them in a similar manner as to human decision-makers (Barocas et al., 2019). In the case of hiring decisions, with or without algorithmic support, *non-discrimination law* (European Council, Directive 2000/43/EC, 2000; European Council, Directive 2000/78/EC, 2000; European Parliament and the Council, Directive 2006/54/EC, 2006) and *data protection law* [European Parliament and the Council, Regulation (EU) 2016/679, 2016] in particular must be observed (Hacker, 2018). RS users, in addition, should also take note of newly adopted laws regulating the online realm and AI technology in the EU.^11^ Most notably in this context are the Digital Markets Act [European Parliament and the Council, Regulation (EU) 2022/1925, 2022] and the Digital Services Act [European Parliament and the Council, Regulation (EU) 2022/2065, 2022] which are already in force, as well as the Proposal for an Artificial Intelligence Act (European Commission, Proposal Artificial Intelligence Act, 2021) which could become law within 2024. While ordinarily the term “fairness” is used to describe fair and equal divisions of resources, in law the concept dealing with this in the world of employment is “non-discrimination.” The fundamental right of non-discrimination is highlighted in all of the recent EU regulations. In the specific case of RRSs, by virtue of being applied in recruitment, the algorithmic decision has to comply with the directives of non-discrimination law.

Compared to these legal sources and terminologies, researchers and practitioners in computer science (CS) and artificial intelligence (AI) commonly use the term “fairness” and quantify it according to some computational metric, as we have seen in Sections 2.3 and 2.4. Therefore, their assumption is that a system can be more or less fair. In stark contrast, the legality of a decision cannot be measured, it is either a case of discrimination or it is not. The benchmark is non-discrimination law. A system cannot in principle base its hiring decision on the sex, race or ethnicity, religion or belief, disability, age, or sexual orientation of the candidates, as EU law prohibits unjustified differential treatment on the basis of these protected characteristics (direct discrimination). A system can also not use an apparently neutral criterion which will have the effect of disadvantaging a considerably higher percentage of persons sharing the protected characteristic (indirect discrimination). This would happen if a system is applied in the same way to everybody, but disadvantages a group of people who share a protected characteristic.

The legal literature, despite the terminological incongruence, has picked up the most used fairness definitions in CS and AI research and often categorized algorithms that adopt them as either blind (or unaware) algorithms or protected-characteristics-aware algorithms (Žliobaitė and Custers, 2016; Bent, 2019; Wachter et al., 2020; Kim, 2022). The former describes algorithmic designs that achieve their results without using any of the protected characteristics as grounds for decision. The algorithm is, therefore, not given any information about any of the protected attributes. The latter describes algorithms which use some or all protected characteristics in their data to make decisions. Blind algorithms not only tend to underperform (Žliobaitė and Custers, 2016; Bent, 2019; Xiang and Raji, 2019; Wachter et al., 2020; Kim, 2022), but also often learn spurious correlations in the data that can serve as proxies for the protected characteristics in their datasets, which can equally lead to discrimination cases (Žliobaitė and Custers, 2016; Chander, 2017; Kim, 2017; Bent, 2019; Wachter et al., 2020; Adams-Prassl, 2022; Hildebrandt, 2022).^12^ This can occur, for instance, when a correlation between ethnicity and postal code is drawn or when the recommendation algorithm unintentionally incorporates implicit gender information from interaction data because of different preferences of male and female users (Ganhör et al., 2022). The fairness definitions presented above all use protected characteristics to achieve fairness (Barocas et al., 2019; Bent, 2019).

### 3.1. Are debiased candidate recommenders affirmative action measures?

Debiased candidate recommenders essentially re-rank candidates according to the fairness definition used in order to achieve a parity for groups with and without certain protected characteristics. Job recommenders inherently also perform a re-ranking, however of job advertisement and employment opportunities. The discrimination risks thereby are similar, albeit center more on the delivery system (e.g., targeted ads). A closer investigation though transcends the scope of this survey (Greif and Grosz, 2023). Affirmative action schemes, known as “positive action” in EU law, are measures by which specific advantages are given to the underrepresented groups in order to compensate for existing disadvantages in working life to ensure full equality. Quotas are the most used example and directly relate to what RRSs are doing by re-ranking candidates. “Fair” algorithmic affirmative action, as strived for by the fairness definitions, does however not translate to a lawful understanding of the concept (Xiang and Raji, 2019). The Court of Justice of the EU (CJEU) has previously stated that schemes which give “absolute and unconditional priority” exceed the limitations of the positive action exception (CJEU C-450/93, 1995). In subsequent case-law, the CJEU however narrowed the scope explaining that flexible quotas allowing for individual consideration would be in line with the positive action exception (CJEU C-409/95, 1997). As long as a “saving clause” is provided for, allowing for an objective assessment of all criteria, which can “override the priority accorded [...] where one or more of those criteria tilts the balance in favor of [another] candidate,” a quota scheme would be line with EU law (CJEU C-409/95, 1997). Thus, every case involving the hiring according to a quota scheme must be open for individual consideration. Whether this could be computable is doubtful (Hacker, 2018; Adams-Prassl, 2022). Any candidate recommender re-ranking candidates to achieve a fixed fairness definition without taking each candidates' individual circumstances into account will most likely run afoul of the legal requirements set out. For instance, imagine an employer deciding between two candidates, A and B, to fill one position. A and B are materially equally qualified, meaning that there resumes, though not necessarily the same, are of equivalent value for this position. Candidate A should be hired according to the affirmative action scheme in place. The employer however should (according to the “savings clause”) still hire candidate B, if there are individual circumstances, reasons specific to that candidate (e.g., sole provider or long term unemployed), which tilts the balance in candidate B's favor. This needs to be decided on an individual case level and is therefore hard to automatize in a RRSs. This, however, must not mean the end of all algorithmically assisted hiring.

### 3.2. Pre-, in-, or post-processing: walking the legal line

As Hacker (2018) notes, the case-law dealing essentially with “corrective powers” at or after the selection process faces greater scrutiny from the CJEU than measures applied before. On the other hand, the CJEU seems to be more lenient when positive action measures are applied *before* first selection (CJEU C-158/97, 2000). Indeed, “positive measures” (even strict quotas) before the actual selection stage of the decision procedure are more likely to be accepted by the CJEU (Hacker, 2018). This could make implementing algorithmic fairness during the *training* stage of a model and before actually ranking candidates wrapped as a quota scheme widely applicable (Hacker, 2018; Adams-Prassl, 2022). Approaches including balancing the training dataset or re-ranking of (fictitious) candidates in the training phase of a candidate recommender should in principle be valid options for routing out biases before the model is put on the market. A glance over to the US shows a similar approach: US case-law suggests that rearrangement in terms of affirmative action applied *after* the selection results have been allocated to the respective candidates^13^ could lead to discrimination of the now down-ranked selected candidates (US Supreme Court, Ricci v DeStefano, 2009).

For both jurisdictions (EU and US), the crux is timing: taking into account biases (and potentially discrimination) that is found in society and in datasets is possible (also via algorithmic help), as long as it is done *before* real-world application. Otherwise, a candidate recommender risks producing further discrimination by ranking candidates first and foremost according to a protected characteristic (e.g., sex) and not according to actual suitability for the job in question. Kim (2022) notes additionally that the US Supreme Court acknowledges that an employer may need to take protected characteristics into account to create fairer hiring processes. Along with Bent (2019), Kim (2017) argues that this should leave room for algorithmic affirmative action before the selection process. Therefore, as it stands, in terms of (both EU and US) non-discrimination law, pre-processing approaches should be favored, whereas post- and probably in-processing approaches would most likely run afoul of current requirements.

## 4. Conclusions and open challenges

In this survey, we analyzed the current fairness research in RRSs from multiple perspectives. First, the algorithms used in RRSs were classified into four categories. Subsequently, we consolidated different fairness definitions in the existing literature to understand the objectives of fairness research in RRSs. We provided the fairness definitions used in classification and connected them with their adapted forms for RRSs. Further, we detailed some of the fairness metrics found in the surveyed literature. We also discussed the work done to analyze the presence or absence of fairness in RRSs. Subsequently, the most common pre-, in-, and post-processing approaches to gain fairness in RRSs were described. Finally, we bridge the gap to legal scholarship by discussing fairness definitions and their relation to legal requirements in order to provide an overview of some of the possible legal issues resulting from unfairness in RRSs. We thereby identified the lack of interdisciplinary vocabulary and understanding as a substantial challenge.

RRSs is a quickly evolving field but is also facing several challenges and open questions which are waiting to be solved. First, the *lack of public datasets for fairness research* in the recruitment domain is evident, as highlighted in Table 2. The usage of private data and proprietary RRSs limits the understanding and reproducibility of fairness research.

Experiments are most often *limited to gender* as sensitive attribute. Other attributes such as ethnicity, age, or disability are commonly only targeted when studied in conjunction with gender and are often modeled artificially. This limits our understanding of the role of non-gender attributes in the recruitment domain. At the same time, it is exciting to see the recent works considering discrimination against groups defined by not only a single attribute but a combination of attributes.

Fairness definitions are also an increasing challenge for the community. They are most commonly *adapted from classification fairness definitions* for RSs. The adapted definitions for job recommenders does not consider the ranks of recommended items. This, however, is important for both candidate and job recommenders. Individual fairness (Markert et al., 2022) is an exciting new direction away from the standard of group-associated fairness definitions such as demographic parity and equal opportunity.

The fairness metrics are highly diverse across the literature surveyed. We would like to highlight here that even when the fairness definitions targeted are the same, the metrics used for fairness measurement are rarely identical. This points to the problem of a *lack of standardized fairness evaluation metrics* in the recruitment domain.

The recruitment domain is a content-rich domain (i.e., resumes and job posts both convey lots of descriptive textual semantics), which explains the prevalence of CBF and KB recommendation algorithms. The *scarcity of hybrid and CF algorithms* in RRSs research on fairness shows a disconnect with common RSs research (de Ruijt and Bhulai, 2021). In addition, while currently *post-processing approaches are most often adopted* for debiasing, as discussed in Sections 2.6, 3.2, they are also the most concerning ones from a legal perspective. We, therefore, suggest to devote more research to pre-processing and in-processing strategies. In addition, we strongly advocate for more interdisciplinary research, involving experts from both RSs and legal scholarship, to formulate strategies and constraints for legally suitable in- and post-processing approaches and to drive RS research in the recruitment domain.

To wrap up, the surveyed research works are (1) diverse in terms of job/candidate recommenders and the adopted algorithms, (2) explore new fairness definitions such as IF, and (3) experiment with various metrics that are attempts to better represent the same fairness concept. The current trajectory of fairness research in RRSs is highly promising, but several avenues for further improvements through the valuable input from diverse research communities is required.

## Author contributions

Conceptualization and funding acquisition: MS, EG, and NR. Literature search and writing—original draft: DK and TG. Writing—review and editing: MS, NG, and EG. All authors approved the submitted version.

## Footnotes

^1^https://dblp.org/ (accessed May 2023).

^2^https://www.indeed.jobs/, https://www.monster.com/, https://www.careerbuilder.co.uk/ (accessed May 2023).

^3^https://www.taskrabbit.com/, https://jobs.google.com/about/ (accessed May 2023).

^4^https://www.kaggle.com/competitions/job-recommendation/overview (accessed June 2023).

^5^https://commoncrawl.org/ (accessed June 2023).

^6^https://www.kaggle.com/datasets/davinwijaya/employee-turnover (accessed June 2023).

^7^https://www.kaggle.com/datasets/liujiaqi/hr-comma-sepcsv (accessed June 2023).

^8^The actual protected group is not mentioned by the creators.

^9^https://textio.com/ (accessed May 2023).

^10^https://www.pymetrics.ai/ (accessed May 2023).

^11^https://digital-strategy.ec.europa.eu/en/policies/european-approach-artificial-intelligence (accessed May 2023).

^12^Proxy discrimination most often comes in the form of indirect discrimination.

^13^In the case of RRSs, this means that candidates have received their score from the system, based on which the ranked list is created.
